# A systematic quantification of hemodynamic differences persisting after aortic coarctation repair

**DOI:** 10.3389/fbioe.2025.1539256

**Published:** 2025-06-26

**Authors:** Christopher Jensen, Arash Ghorbannia, David Urick, G. Chad Hughes, Amanda Randles

**Affiliations:** ^1^ Department of Biomedical Engineering, Duke University, Durham, NC, United States; ^2^ Division of Cardiovascular and Thoracic Surgery, Department of Surgery, Duke University Medical Center, Durham, NC, United States; ^3^ Department of Radiology, Duke University Medical Center, Durham, NC, United States

**Keywords:** aortic coarctation, computational fluid dynamics, late aneurysmal degeneration, restenosis, long-term complications of coarctation

## Abstract

**Introduction:**

Aortic coarctation (CoA) comprises 6%–8% of all congenital heart diseases and is the second most common cardiovascular disease requiring neonatal surgical correction. However, patients remain at high risk for long-term complications, notably recoarctation.

**Methods:**

Hemodynamic simulations were performed in a group of six patients following CoA repair, as compared to a group of age and sex-matched healthy controls. Progressive narrowing at the CoA repair site was modeled to simulate the recoarctation process. Key measurements included time-averaged wall shear stress (TAWSS) in the aortic arch and CoA repair site.

**Results:**

Repaired aortas demonstrated significantly higher TAWSS compared to healthy aortas in the aortic arch (3.46 vs 1.24 Pa, *p* < 0.05) and CoA repair site (4.34 vs 1.56 Pa, *p* < 0.05). A pronounced nonlinear relationship between stenosis severity and TAWSS was observed suggesting that increasing stenosis corresponds to progressively abnormal shear stress.

**Discussion:**

The persistent high TAWSS in CoA-repaired aortas may underlie the poor long-term outcomes observed in this population. The identified nonlinear relationship between stenosis severity and TAWSS magnitude suggests a potential positive feedback mechanism, where abnormal shear stress exacerbates pathologic remodeling in the repaired aorta, highlighting the potential role of hemodynamic simulations in the clinical management of CoA patients.

## 1 Introduction

Aortic coarctation (CoA) is a congenital cardiovascular disease characterized by a narrowing of the distal aortic arch and/or proximal descending aorta. Left untreated, patients with CoA are at high risk of long-term complications such as severe hypertension, aortic aneurysms, congestive heart failure, and early death (Rachel, 2015; Kim et al., 2020). As such, clinicians have developed a variety of surgical repairs that attempt to restore normal aortic anatomy, hemodynamics, and end-organ perfusion (Baumgartner et al., 2020; Chetan and Mertens, 2022). Complete resection with end-to-end anastomosis (REEA) is associated with some of the best long-term outcomes, with 10-year survival exceeding 90% (Gropler et al., 2019; Lehnert et al., 2019; Choudhary et al., 2015). However, even after a best-case CoA repair, up to 50% of patients will still develop a serious complication at the repair site such as restenosis (Dias et al., 2020; Cramer et al., 2013; Hoffman et al., 2014), which often requires reoperation and is associated with increased long-term mortality and morbidity (Celermajer and Greaves, 2002; de Divitiis et al., 2005; O’Sullivan et al., 2002; Ou et al., 2004). Consequently, lifetime surveillance imaging is recommended for all patients following CoA repair, since clinicians cannot yet predict which individuals are going to ultimately develop recoarctation requiring reoperation (Dias et al., 2020; Stout et al., 2018). This underscores the need for improved risk stratification and monitoring to reduce long-term morbidity.

Prior clinical research has already identified multiple risk factors for long-term restenosis, such as younger age and/or lower birth weight at the time of initial repair (McElhinney et al., 2001; Lehnert et al., 2019; Truong et al., 2014; Burch et al., 2009; Hager et al., 2008), the presence of a hypoplastic aortic arch (Hage et al., 2007; Dias et al., 2020), high postoperative flow velocities (Truong et al., 2014), and the choice of initial balloon angioplasty over surgical repair (Chiu et al., 2013; Sen et al., 2018). As many of these predictors are either explicitly anatomic (CoA severity, choice of surgical repair) or directly linked to anatomy (elevated flow velocities), a natural question arises: do residual anatomic abnormalities, particularly at the repair site, lead to postoperative hemodynamic environments that predispose patients to restenosis after CoA repair?

This hypothesis is supported by recent research demonstrating that residual disturbances in endothelial wall shear stress (WSS), often due to anatomic anomalies resulting from CoA or surgical resection, persistently trigger mechanotransduction pathways that mediate pathologic aortic remodeling (Paukner et al., 2024; Ghorbannia et al., 2024; Ghorbannia et al., 2023). Therefore, characterizing and quantifying the relationship between postoperative aortic anatomy and hemodynamic metrics such as WSS after CoA repair could allow clinicians to better anticipate an individual patient’s long-term risk of restenosis and preemptively screen high-risk patients more closely. However, traditional clinical outcomes research and *in vivo* animal models are not well-suited for understanding, analyzing, and comparing patient-specific hemodynamic environments; an alternative approach is necessary.

In recent years, computational fluid dynamics (CFD) has become an increasingly popular tool for investigating patient-specific hemodynamics in a wide range of cardiovascular diseases, including CoA (Gerrah and Haller, 2020; Marelli et al., 2022). Specifically, CFD simulations allow researchers to study metrics of bulk blood flow (including commonly used clinical parameters such as blood flow rate and pressure), as well as local hemodynamic metrics such as endothelial WSS, which are not clinically measurable but are clearly linked to vascular remodeling and patient physiology. While early CFD studies were primarily characterizing flow patterns in (semi)-idealized CoA models (Keshavarz-Motamed et al., 2013; Gounley et al., 2016a), the proliferating interest in this topic has led to progressively anatomically and physiologically accurate patient-specific models that can now accurately identify individual patients with hemodynamically-significant CoA requiring intervention (Lu et al., 2020; Nair et al., 2024).

While many CFD studies of CoA have been completed in recent years, it is difficult, if not impossible, to draw a consistent conclusion from their results. These investigations employ a diverse range of aortic geometries, boundary conditions, computational techniques, and reported hemodynamic metrics (Aslan et al., 2020; Guillot et al., 2019; Olivieri et al., 2011; Goodarzi Ardakani et al., 2022; Keshavarz-Motamed et al., 2016; Keshavarz-Motamed et al., 2013; Gounley et al., 2016a; Nicole Antonuccio et al., 2021; Mariotti et al., 2023). This heterogeneous study design significantly complicates any attempt to develop a consistent picture of the interplay between patient anatomy, surgical repair strategy, cardiovascular physiology, aortic hemodynamics, and clinically relevant outcomes such as long-term restenosis risk. For example, if we take a common, CFD-specific metric such as WSS and simply ask, “How does WSS change at the coarctation site following repair?“, we would find answers running from the gamut from “WSS increases” (Caimi et al., 2020; Keshavarz-Motamed et al., 2013; Olivieri et al., 2011; Rafieianzab et al., 2021) to “WSS decreases” (Goodarzi Ardakani et al., 2022; LaDisa et al., 2011a; LaDisa et al., 2011b; Gounley et al., 2016a) to “it depends” (Keshavarz-Motamed et al., 2016; Sadeghi et al., 2020), to not even being reported as an outcome (Aslan et al., 2020; Guillot et al., 2019).

The purpose of this study is to isolate and quantify the influence of postoperative anatomy (*i.e.*, the extent of surgical repair and residual stenosis) on aortic endothelial wall hemodynamics. First, we directly compare the hemodynamic environments of a group of CoA patients following identical surgical repairs with a cohort of age- and sex-matched control patients with normal aortas. Next, we synthetically generate different levels of residual CoA stenosis to model the influence of residual/progressive stenosis on postoperative hemodynamics. Throughout this process, we make significant efforts to standardize, normalize, and quantify our reported findings in order to develop a clear and consistent picture of the relationship between aortic anatomy, residual lesion severity, and postoperative hemodynamics following CoA repair. We hope this work will guide and/or motivate future CFD models designed to predict an individual patient’s risk of restenosis following CoA repair, thereby allowing clinicians to develop personalized surveillance and treatment plans for these patients.

## 2 Methods

### 2.1 Patient selection

**FIGURE 1:**
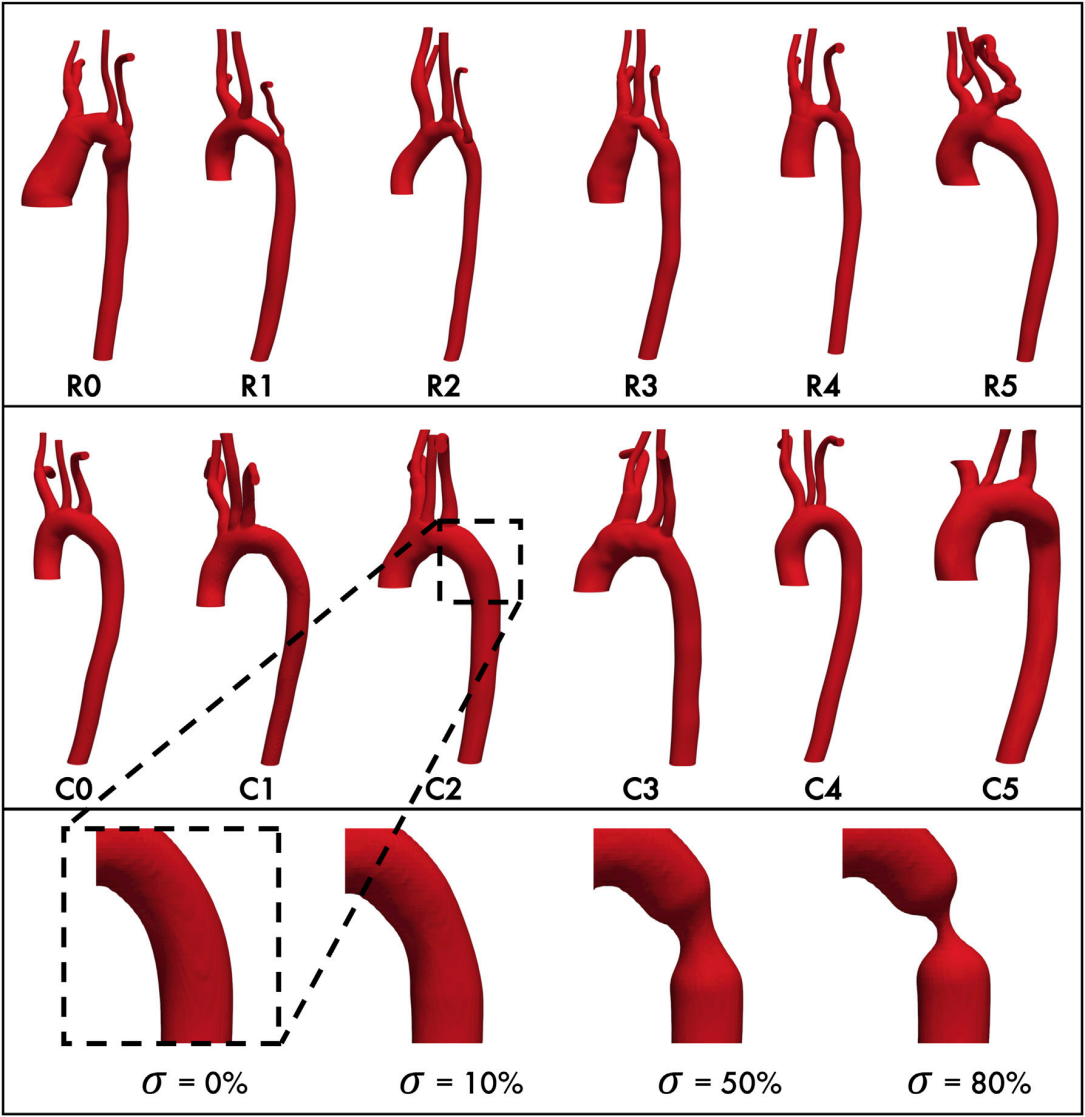
Baseline aortic geometries of repaired coarctation patients (**top row**) and healthy control patients (**middle row**.) Each geometry is paired with its matched control (*e.g.*, R0 with C0, R1 with C1, etc. **Bottom row, from left**: Representative geometry with stenosis σ=0 % (unmodified baseline), 10%, 50%, and 80%.

Patient-specific aortic geometries derived from MRI angiograms were obtained from the Vascular Model Repository (VMR), an open source database developed for clinically-motivated CFD investigations (Pfaller et al., 2022). We selected 12 patient aortas for simulation: six from patients after CoA repair REEA (“Repair” cohort, Figure 1, top), and six from age- and sex-matched control patients with healthy, anatomically normal aortas (“Control” cohort, Figure 1, middle). Each geometry consisted of the ascending aorta, the aortic arch/associated branch vessels (brachiocephalic, left common carotid and left subclavian arteries) and the descending aorta, terminating proximal to the iliac bifurcation. In order to only compare patients with similar aortic anatomies, we excluded patients who either 1) underwent CoA repairs other than REEA or 2) had collateralized arterial networks between the arch vessels and descending aorta, (*e.g.*, Patient #3 in this sampling of different CoA variants (LaDisa et al., 2011b)). Since all data were anonymous and open source, this study did not require IRB approval or informed consent.

### 2.2 Generating aortic geometries

The aortic geometries of all 12 study patients were downloaded from the VMR database as stereolithography (STL) files. We will refer to these unmodified, anatomically-accurate, patient-specific geometries as the “baseline” geometries/models for the remainder of this study. Since we also wanted to model the interaction between aortic endothelial shear stress and the restenosis process, we then modified each baseline geometry to have progressively severe concentric stenoses of 10%, 50%, and 80% (Figure 1, bottom) at the CoA repair site (for the Repair cohort) or at the anatomically corresponding location immediately distal to the left subclavian artery where re-stenosis is most often seen (for the Control cohort). This resulted in a total of 48 aortic geometries for this study.

Geometry modifications were performed with the open source packages morphMan (version 1.4, Simula Research Lab, Norway) (Bergersen et al., 2020) and Blender (version 2.7.9, Amsterdam, Netherlands) where Voronoi diagrams derived from the discretized surface mesh were created to represent the geometry in terms of centerlines, maximum inscribed spheres, and their radius. Then, the inscribed spheres and the corresponding radius were scaled relative to the centerlines in the regions of interest to circumferentially “pinch” the aortic wall, thereby recreating a restenosis in the descending thoracic aorta distal to the left subclavian artery (Figure 1). Consistent with previous definitions (Ou et al., 2004; Donazzan et al., 2014; Ou et al., 2008), the severity of the restenosis lesion σ was defined as:

$$\sigma =1-\frac{R_{\mathrm{stenosis}}}{R_{\mathrm{baseline}}}$$ (1)

where $R_{\mathrm{stenosis}}$ is the aortic diameter at the most severe point of stenosis, and $R_{\mathrm{baseline}}$ is the corresponding baseline aortic diameter at that location. Modified geometries (in both cohorts) will be referred to by the degree of stenosis (sigma) introduced, as defined above in Equation 1.

### 2.3 Computational hemodynamics modeling

Fluid simulations were performed with HARVEY (Peters Randles et al., 2013), a massively parallel CFD code that uses the lattice Boltzmann method (LBM) to solve the Navier-Stokes equations of fluid flow. A comprehensive description of the LBM methodology applied to fluid dynamics can be found elsewhere (Succi, 2003), and HARVEY’s development and validation for simulating cardiovascular flow have been previously described (Randles et al., 2015a; Randles et al., 2015b; Feiger et al., 2019; Vardhan et al., 2024). Briefly, instead of directly solving for fluid velocity and pressure, LBM-based solvers such as HARVEY take an alternative approach of modeling fluid as a probability distribution f(x,t) of particles within a discrete 3D Cartesian lattice. The temporospatial evolution of this particle distribution over a specified time interval Δt is governed by collision interactions described by the lattice Boltzmann equation (Equation 2), where Ω represents the collision operator and $f_{i}^{eq}\left(x,t\right)$ represents the particle distribution at equilibrium:

$$f_{i}\left(x+c_{i}\Delta t,t+\Delta t\right)=f_{i}\left(x,t\right)-\Omega \left(f_{i}\left(x,t\right)-f_{i}^{eq}\left(x,t\right)\right)$$ (2)

HARVEY uses a standard D3Q19 velocity discretization pattern and a single-relaxation Bhatnager-Gross-Krook (BGK) collision operator. Due to the high shear rate $\left(>100\;s^{-1}\right)$ present in large arteries (Alexy et al., 2022), the blood is modeled as an incompressible Newtonian fluid with constant density $\rho =1,060\;kg/m^{3}$ and dynamic viscosity μ=0.004Pa⋅s , with a no-slip boundary condition enforced at the fluid-wall interface using a halfway bounce-back method. All vessel walls are assumed to be rigid, which is commonly done in fluid simulations of large vessel blood flow (Goubergrits et al., 2015; LaDisa et al., 2011a; LaDisa et al., 2011b).

### 2.4 Boundary conditions and hemodynamic metrics

We wanted our simulations to best isolate the effect of vessel anatomy and CoA lesion severity σ on aortic hemodynamics. Consequently, to minimize the risk of confounding the results by implementing different boundary conditions for each simulation to achieve a physiological state selected *a priori*, we chose to apply simple and consistent boundary conditions to all simulations. To capture time-averaged hemodynamic metrics such as TAWSS and OSI, pulsatile blood flow at the aortic inlet was modeled as a time-varying velocity waveform with a period *T* of 0.75 s (corresponding to a heart rate of 80 beats per minute), a maximum systolic velocity of 0.45 m/s, and a parabolic wave profile consistent with previous studies using the same repository (LaDisa et al., 2011a; LaDisa et al., 2011b). The outlets were modeled with 0-pressure boundary conditions. Spatial convergence studies were performed at grid spacings of 15, 20, 25, 50, and 75 microns, with convergence results seen for all simulations at a resolution of 25 microns. Specifically, maximum values of TAWSS and OSI were monitored at the CoA region for incrementally decreasing grid spacings until ≤3% change was observed which was the case for all the simulations done at grid spacings <50 microns. Temporal convergence studies were performed for a duration of seven cardiac cycles, with simulation stability observed after the second cardiac cycle.

The primary outcomes of interest were endothelial WSS τ(x,t) , TAWSS (Equation 3), and OSI (Equation 4):

$$TAWSS=\frac{1}{T}\int_{0}^{T}|\tau \left(t\right)|\;dt$$ (3)

$$OSI=\frac{1}{2}\left(1-\frac{\left|\int_{0}^{T}\tau \left(t\right)\;dt\right|}{\int_{0}^{T}|\tau \left(t\right)|\;dt}\right)$$ (4)

These metrics were chosen based upon previous literature linking TAWSS/OSI to abnormal aortic remodeling and to allow direct comparison with the results of previous CFD investigations of CoA.

### 2.5 Framework for standardized aortic comparisons

**FIGURE 2:**
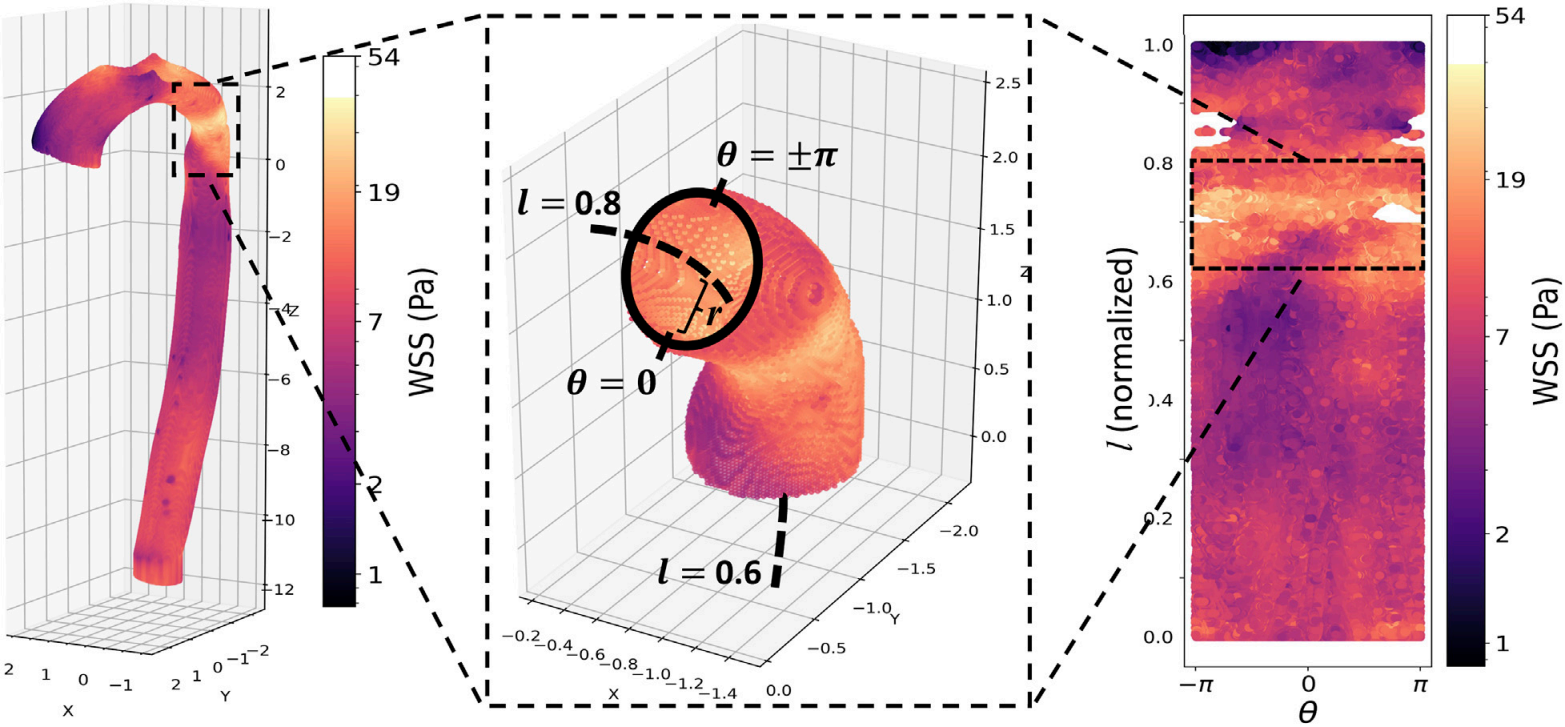
**Top panel, from left**: Peak systolic wall shear stress (WSS) in the aorta of a patient with repaired CoA. The repair site is clearly visible as a band of elevated WSS. **Middle**: a vessel wall section demonstrating the cylindrical coordinate re-parameterization: *r* is the distance from the aortic wall to the vessel centerline (dashed line); θ=0 corresponds to the inner curve of the aorta, while θ=±π corresponds to the outside curve; finally, the vessel centerline is normalized to have length 1, ranging from *l* = 1 (aortic inlet) to *l* = 0 (distal aortic outlet). **Right**: This normalized cylindrical coordinate system allows any aortic vessel wall to be “unwrapped” into a 2D plot of *l* and θ , with the repair site now seen as a horizontal band of elevated WSS.

To facilitate comparing simulation results from aortas of different lengths, diameters, and CoA severities σ , all data were spatially normalized prior to analysis. First, every point on the wall of the vessel of each aorta was re-parameterized from Cartesian to cylindrical coordinates that were defined relative to the aortic centerline (Figure 2). For a given vessel wall point P(r,θ,l) , r signifies the shortest Cartesian distance to the vessel centerline, θ represents the circumferential position around the vessel wall (with θ=0 and θ=±π corresponding to the inner and outer curves of the aorta, respectively), and l denotes the normalized position along the vessel centerline, with the aortic inlet set to be l=1 and the distal aortic outlet set to be l=0 . This coordinate transformation, which has been previously employed by other researchers (Perinajová et al., 2021), allows each aortic wall to be unwrapped and assigned to a normalized rectangular two-dimensional domain in θ and l . In turn, this allows us to make quantitative comparisons of the magnitude and spatial distribution of shear metrics within/between cohorts and coarctation severities.

The initial analysis consisted of calculating the circumferentially averaged (*i.e.*, across 0≤θ≤2π ) TAWSS/OSI for each patient. Baseline hemodynamics for the entire cohort were calculated as median [interquartile range] and plotted against the normalized position of the aortic centerline *l*. The aorta was subdivided into five anatomic regions of interest: the ascending aorta (AscAo), the aortic arch, the coarctation repair site (CoA), the proximal descending aorta (pDA) and the distal descending aorta (dDA). Statistical comparisons in each region were performed with the Kolmogorov-Smirnov test for non-normal continuous distributions (Sheskin, 2003). Inter-patient variation in the spatial distribution of TAWSS/OSI was quantified by calculating the L2 norm of the difference in the magnitude of the shear metric, which was then plotted on heat maps.

### 2.6 Accounting for normal inter-patient variation

Since no two patients have anatomically identical aortas, we expected some level of inter-patient variation in shear metrics, even between baseline patient models in the same cohort. Furthermore, these differences would carry over into the σ= 10%, 50%, and 80% variants generated from each baseline geometry. However, we needed to determine that any observed differences in TAWSS/OSI magnitude and spatial distribution between cohorts or different levels of σ were not due to these chance variations in aortic anatomy. Therefore, we needed to account for the variability introduced by these shared features and the interactions between anatomy and hemodynamics.

We did this by using a linear mixed effect (LME) model that incorporates random effects to account for the inherent variation between different patient anatomies (Faraway, 2006). The LME model was employed with the lme4 package in R (Bates et al., 2015). The fixed effect coefficient, as determined from the TAWSS and OSI results, captured the effect of group (control vs repaired) and severity (degree of re-coarctation). The baseline variability was then taken into account by a random intercept. Additionally, the random-effects intercept captures correlations among all measurements derived from a common geometry and can thus be interpreted as a geometry-specific intercept adjustment in the fixed-effects parameter.

We evaluated these factors at each of the five previously defined anatomic regions of the aorta (AscAo, arch, CoA, pDA, and dDA). In addition, we considered angular locations, including the inner, left, outer, and right sides of the aorta. The significant coefficients of the model for the group, severity and their interaction were visualized in Figure 10, with the regions where any of the coefficients were significant highlighted in dark gray.

## 3 Results

### 3.1 Repaired and healthy aortas have significantly different hemodynamics

**FIGURE 3:**
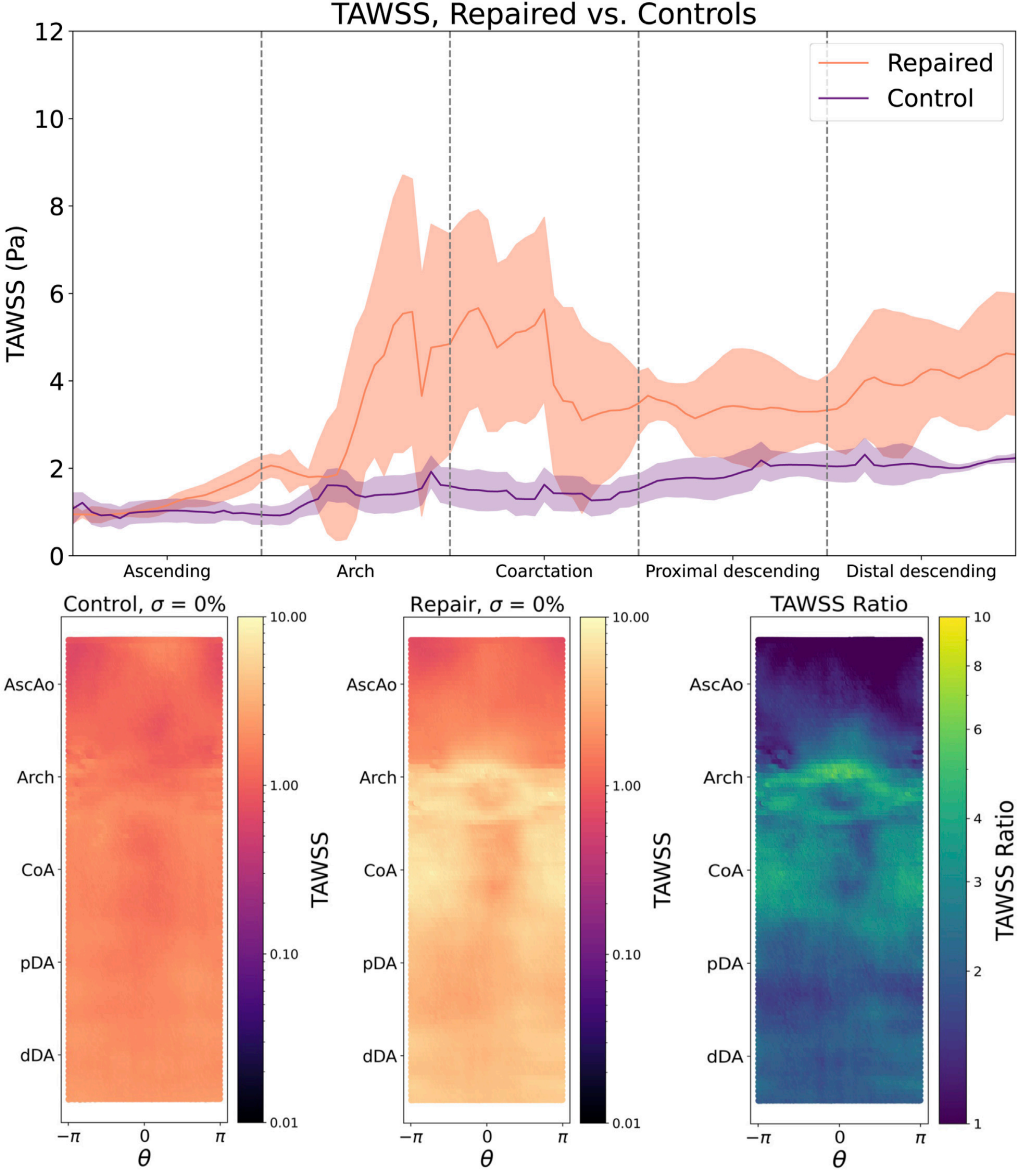
**Top**: TAWSS following CoA repair is significantly elevated in the aortic arch and lesion repair site (bold line = median TAWSS, shaded region = IQR). **Bottom left**: TAWSS is smoothly and uniformly distributed across the aortic endothelium of the control cohort, but is notably elevated around the aortic arch and the outer curvature of the repair site (**bottom middle**), which is further highlighted by the repaired/control TAWSS ratio (**bottom right**).

Simulations showed clear differences in aortic hemodynamics between healthy and repaired patients. The median flow rates in the descending aorta of each patient were 70% of the inlet flow rate, representing a physiologic flow split in all investigated geometries. Healthy patients exhibited a uniform distribution of TAWSS in θ and *l*, which smoothly increased from a median of 1.07 Pa in the AscAo to 2.09 Pa in the dDA (Figure 3, top), due to the slightly narrower diameter of the distal aorta. For repaired patients, the median TAWSS was similar in the AscAo (1.20 Pa) but significantly higher in both the aortic arch (3.46 vs 1.24 Pa, *p* < 0.05) and the CoA repair site (4.34 vs 1.56 Pa, *p* < 0.05, Figure 3, top). This finding is reinforced by heat maps showing that the magnitude of TAWSS is largely isotropic between control patients (Figure 3, bottom left), but in repaired patients it is clearly elevated around the aortic arch and the outer curvature of the coarctation repair site (Figure 3, bottom middle). The elevation of TAWSS along the outer curvature of the aortic and pDA indicates that most repaired aortas exhibit flow separation, with the primary blood flow jet impinging on the distal descending aortic wall, leaving a region of recirculating flow along the inner aortic curvature. This finding is further demonstrated by calculating and plotting the ratio of the magnitude of TAWSS between repaired and control patients (Figure 3, bottom right).

**FIGURE 4:**
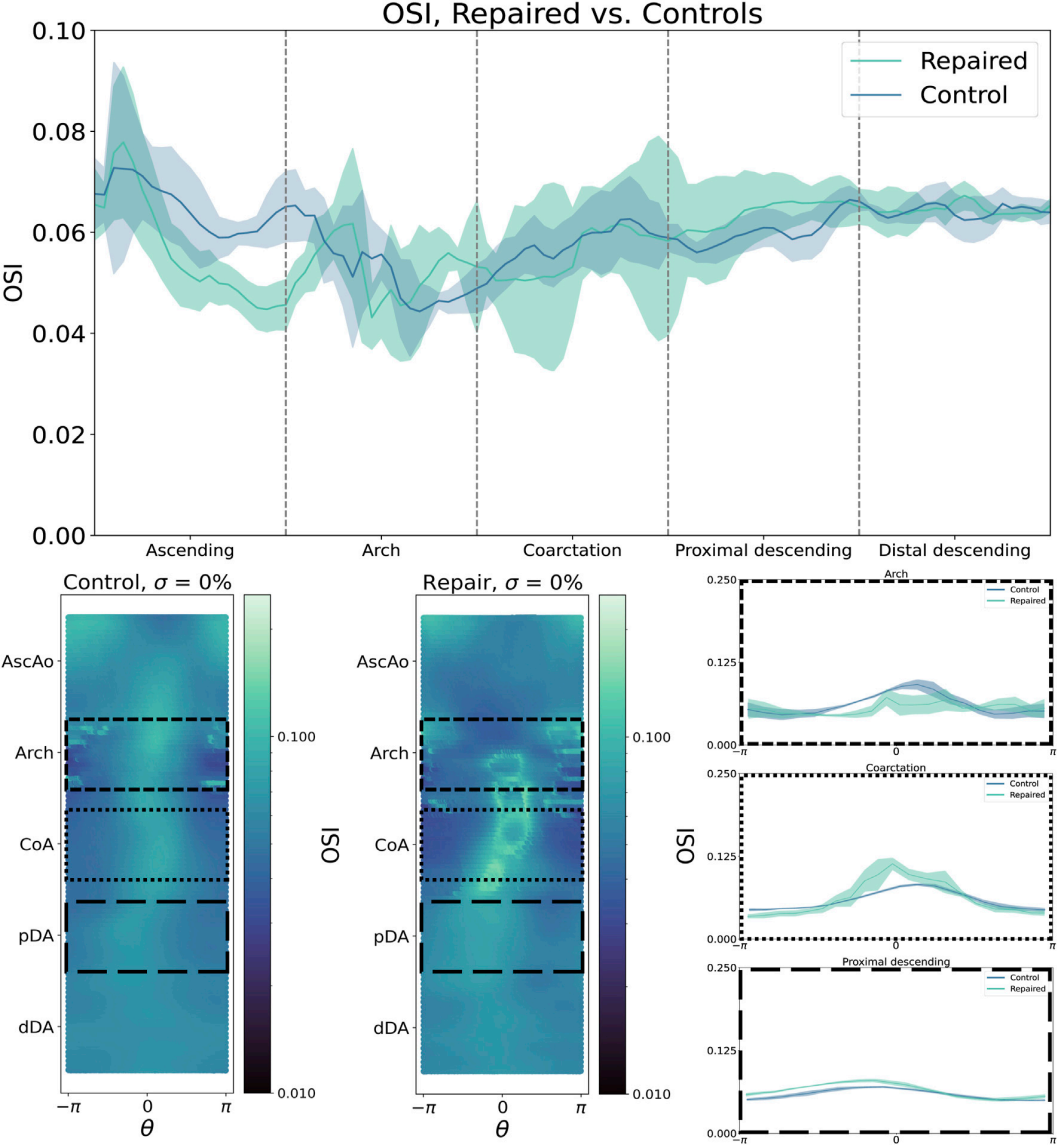
By contrast, oscillatory shear index (OSI) is higher in the aortic arch but lower in the CoA region and pDA. Shear metrics are plotted as median (bold line) with IQR (shaded region).

OSI distributions are consistent with these findings, with control patients showing a smooth, uniform OSI distribution centered around θ=0 , again indicating that normal aortic flow hugs the inner vessel curve from the inlet to the distal aortic outlet (Figure 4, left). In contrast, repaired patients have regions of high OSI centered around θ=0 along the arch and CoA region (Figure 4, bottom center). Together with the TAWSS findings, this pattern of OSI distribution indicates regions of recirculation and low flow along the inner aortic curvature of repaired CoA patients, not strongly pulsatile flow.

### 3.2 Coarctation severity σ strongly influences shear magnitude and distribution

**FIGURE 5:**
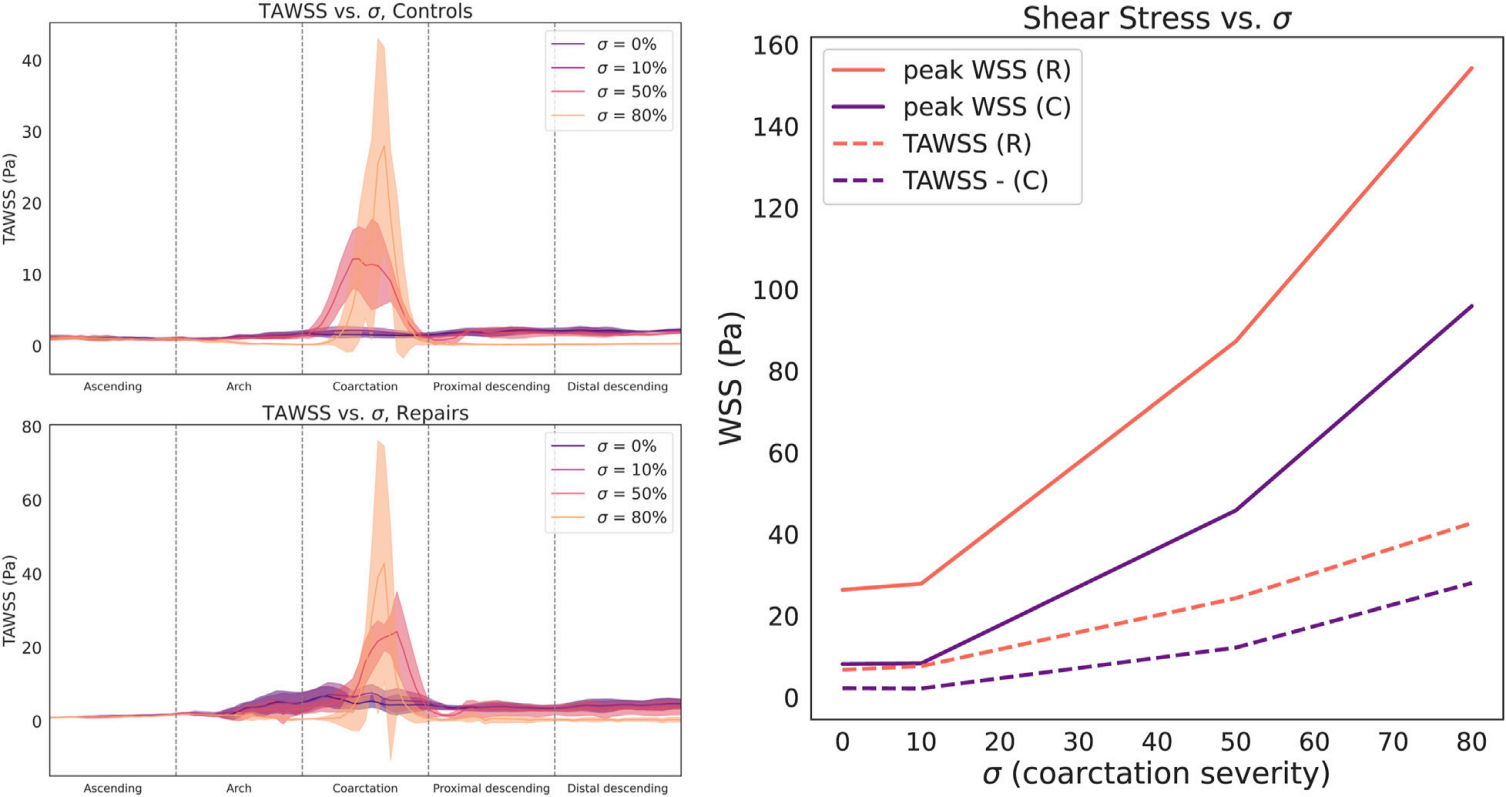
Circumferentially-averaged time-averaged wall shear stress (TAWSS) plotted along the aortic centerline for control (**top left**) and repaired patient cohorts (**bottom left**). Cohort data summarized as median (line) and interquartile range (shaded region). **Right**: Both cohorts demonstrate a sharp, nonlinear relationship between stenosis severity σ and peak TAWSS (dashed lines), as well as peak systolic WSS (solid lines).

We observed a clear, non-linear relationship between CoA severity σ and shear stress magnitude at the CoA site for all investigated geometries (Figure 5). Although TAWSS changed little when increasing σ from 0% to 10%, there was a significant increase from σ=10% to σ=50% (control, 2.17 → 12.19 Pa; repaired, 7.64 → 24.28 Pa), and even more dramatic from σ=50% to σ=80% (control, 12.19 → 27.99 Pa; repaired, 24.28 → 42.70 Pa). The nonlinear increase for peak systolic WSS was even greater (control: 8.33 → 45.81 → 95.89 Pa; repaired: 27.83 → 87.25 → 154.26 Pa).

**FIGURE 6:**
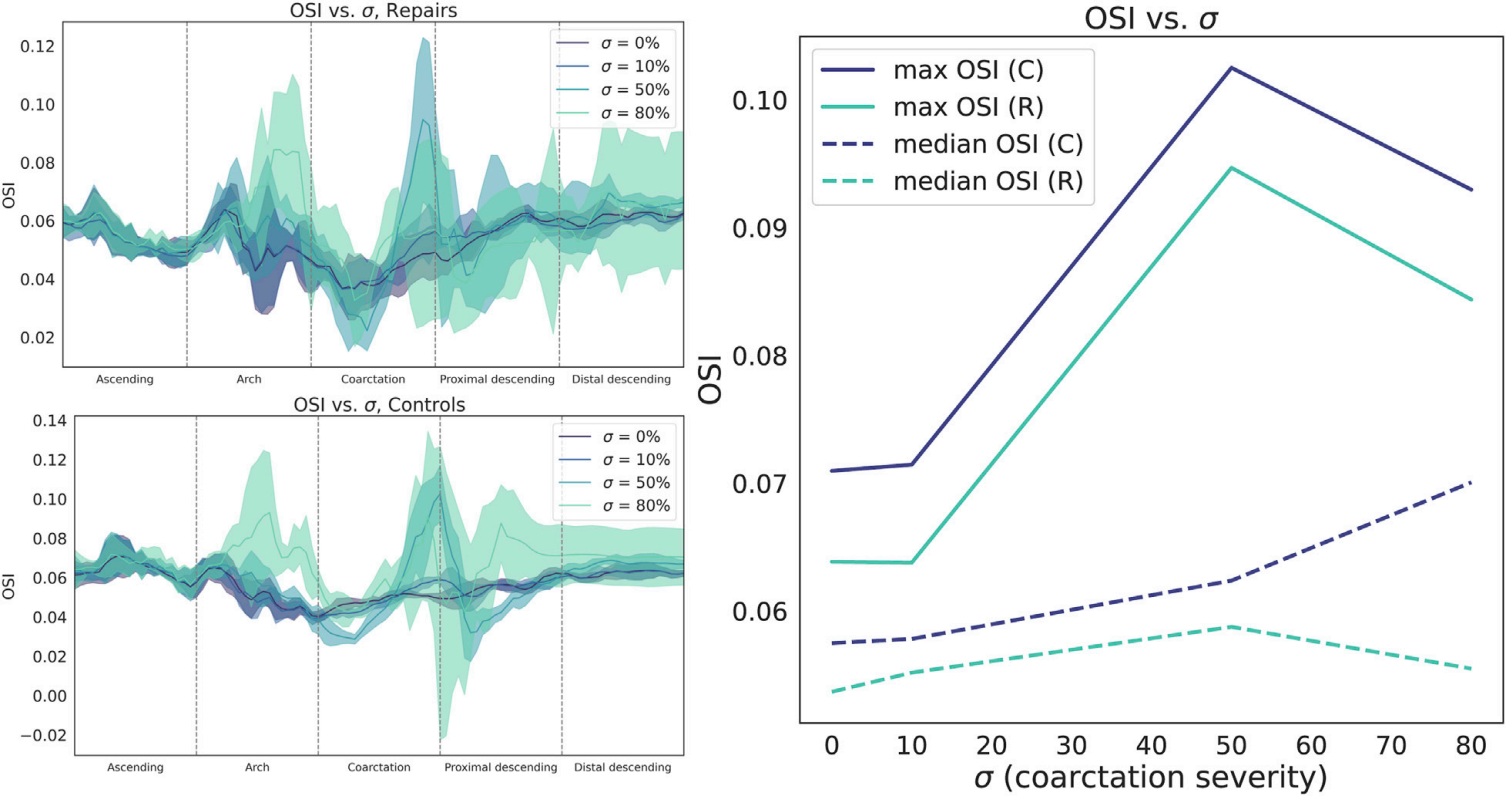
Circumferentially-averaged oscillatory shear index (OSI) plotted along the aortic centerline for repaired (**top left**) and control cohorts (**bottom left**). Cohort data summarized as median (line) and interquartile range (shaded region). **Right**: OSI sharply increases from σ=10 % to 50%, but then decreases.

**FIGURE 7:**
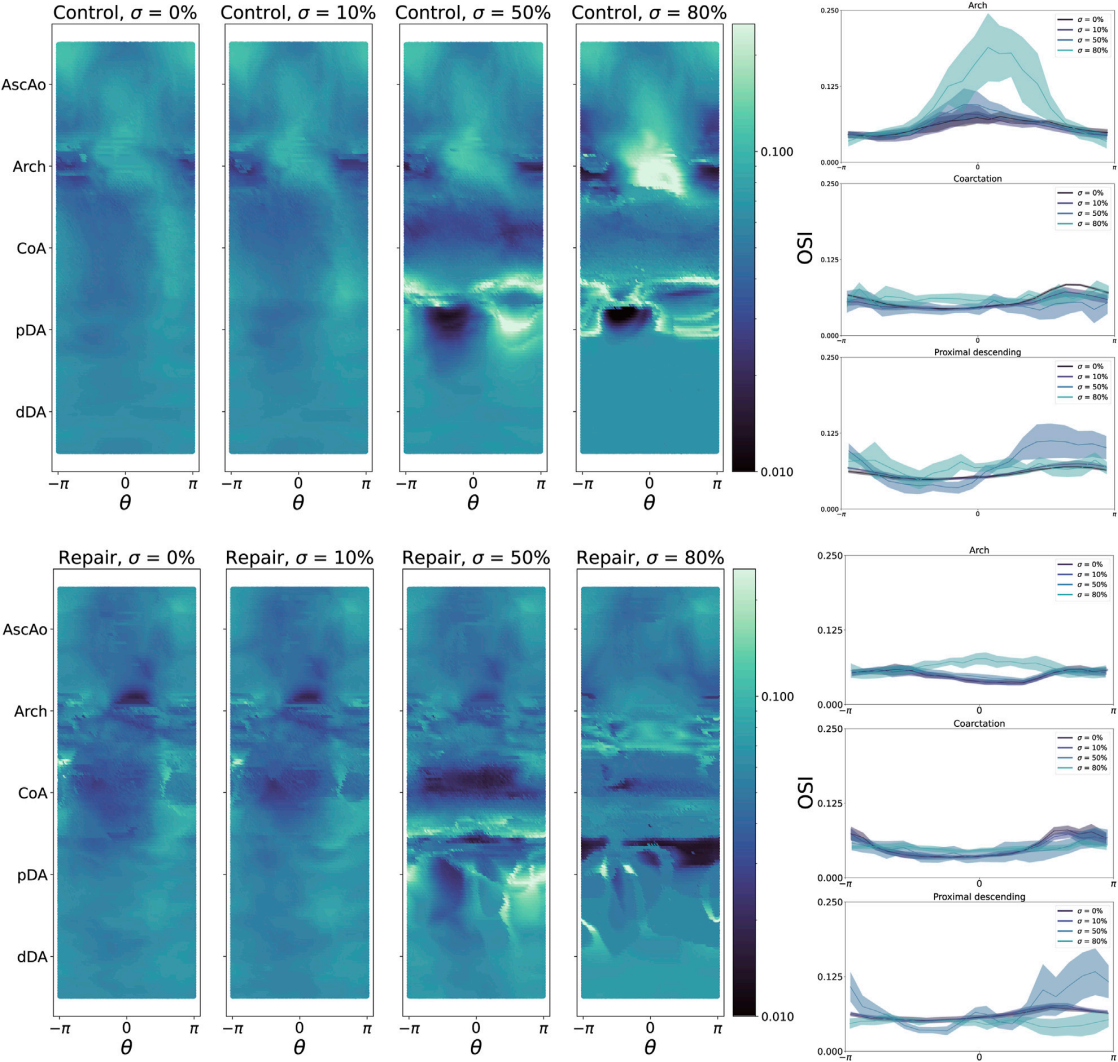
**Left**: oscillatory shear index (OSI) heatmaps for all control geometries (**top**) and repair geometries (**bottom**), stratified by CoA severity σ . **Right, from top**: histograms of OSI distribution across θ at the aortic arch (**top**), coarctation region (**middle**), and proximal descending aorta (**bottom**) can better show OSI variation within aortic regions of interest.

Compared to TAWSS, the relationship between σ and OSI magnitude is more complex (Figure 6). Since OSI can only be calculated over the course of the cardiac cycle, we report the maximum and median OSI within the CoA region. For control patients, increasing σ led to a region of progressively higher OSI in the inner curvature of the AscAo and the outer curvature of the pDA (Figure 7). However, there is no correspondingly clear trend for repaired patients, who demonstrate a weaker trend towards increased aortic arch OSI.

### 3.3 Hemodynamics after CoA repair vary widely

TAWSS distributions in the repaired patient cohort showed considerably more variation than in control patients. For example, the TAWSS IQR was nearly an order of magnitude higher in repaired patients than in control patients (2.92 vs 0.32 Pa, aortic arch, and 1.82 vs 0.20 Pa, repair site). This indicates that a wide range of outcomes are possible after CoA repair, while TAWSS distributions in healthy patients remain fairly uniform.

**FIGURE 8:**
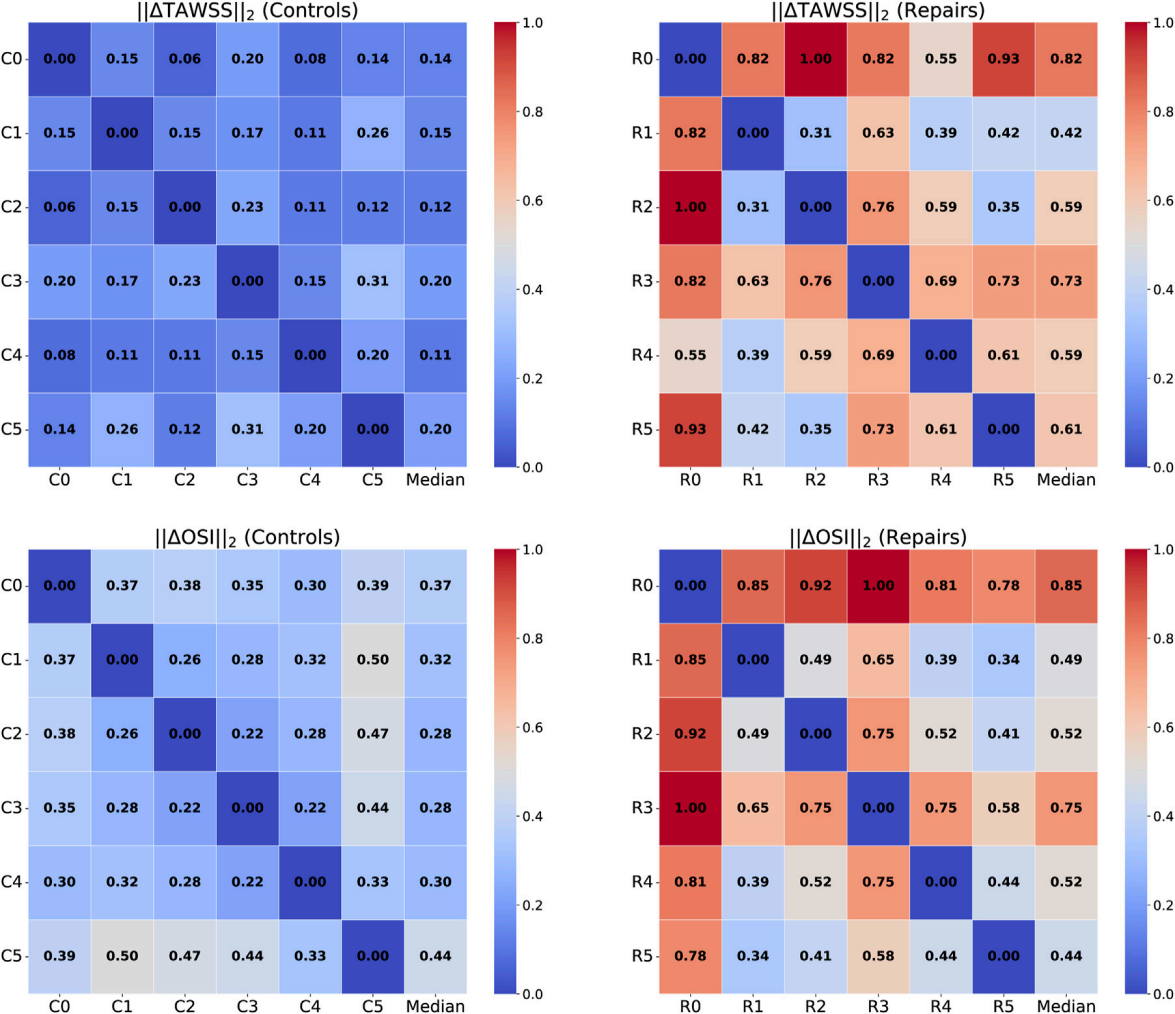
L2 norms of TAWSS (**top row**) and OSI (**bottom row**) for healthy controls (**left**) and repaired patients (**right**). Comparisons are calculated by taking the L2 norm of the difference in TAWSS/OSI between two geometry heatmaps, then normalizing by the maximum L2 norm value. The rightmost column shows the median L2 norm value for each geometry, as compared to all other geometries in the cohort.

**FIGURE 9:**
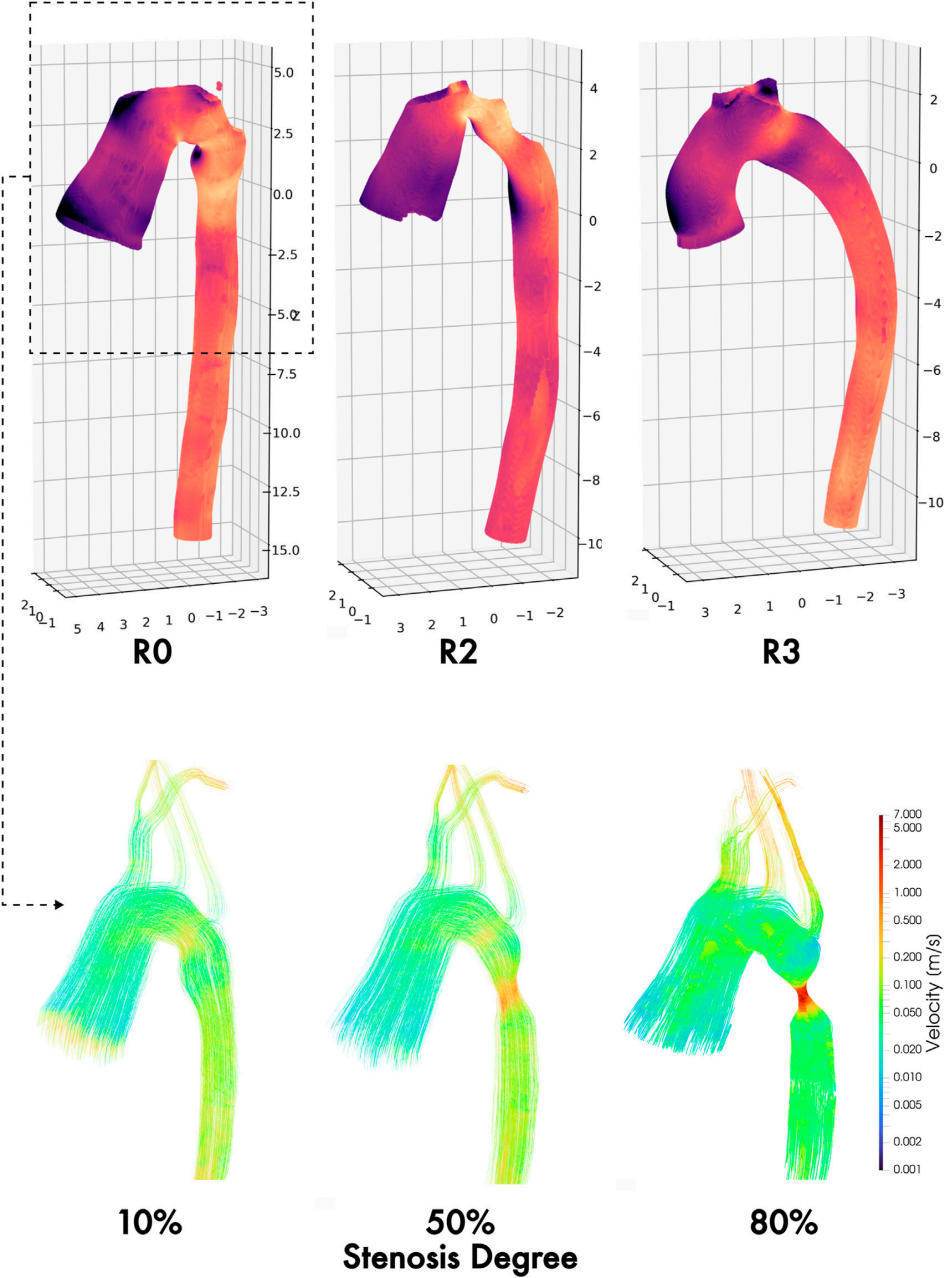
**TAWSS maps** of three different hemodynamic phenotypes following coarctation repair: residual stenosis **(left)**, arch angulation **(middle)**, and normal/uniform **(right)**. **Velocity field streamlines** in the thoracic aortic section at three synthetically generated stenosis degrees (10%, 50%, and 80%), showing progressive complexity of ascending and arch flow regimes by increasing degree of residual and/or re-coarctation.

To further examine this, we quantified intra-cohort variance by calculating the L2 norm of the difference in TAWSS and OSI magnitude between each pair of geometries within each cohort (Figure 8). From this, it is immediately apparent that the hemodynamics between the control geometries are much more similar than between the repaired patients. This technique could be used to potentially identify different “hemodynamic phenotypes” following coarctation repair. For example, in the repaired cohort, the greatest pairwise differences in the TAWSS distribution are between geometries R0, R2, and R3. Plotting these geometries shows clear differences in the TAWSS distribution pattern: R0 shows a high TAWSS mainly around the repair site, while R2 is centered around the narrowed aortic arch, and R3 is qualitatively uniform, although with a slight residual stenosis at the repair site (Figure 9).

### 3.4 Spatial distribution of TAWSS and OSI varies significantly by CoA severity

The implemented LME model distinguished the relative importance of severity or residual stenosis in each studied cohort confirming that aortic coarctation, even after surgical repair, significantly disturbs the spatial distribution of TAWSS and OSI compared to healthy controls. Notably, these differences persist across various regions of the aorta and are not merely a function of the severity of re-coarctation, which is a separate, significant disruptor of TAWSS/OSI patterns in its own right.

**FIGURE 10:**
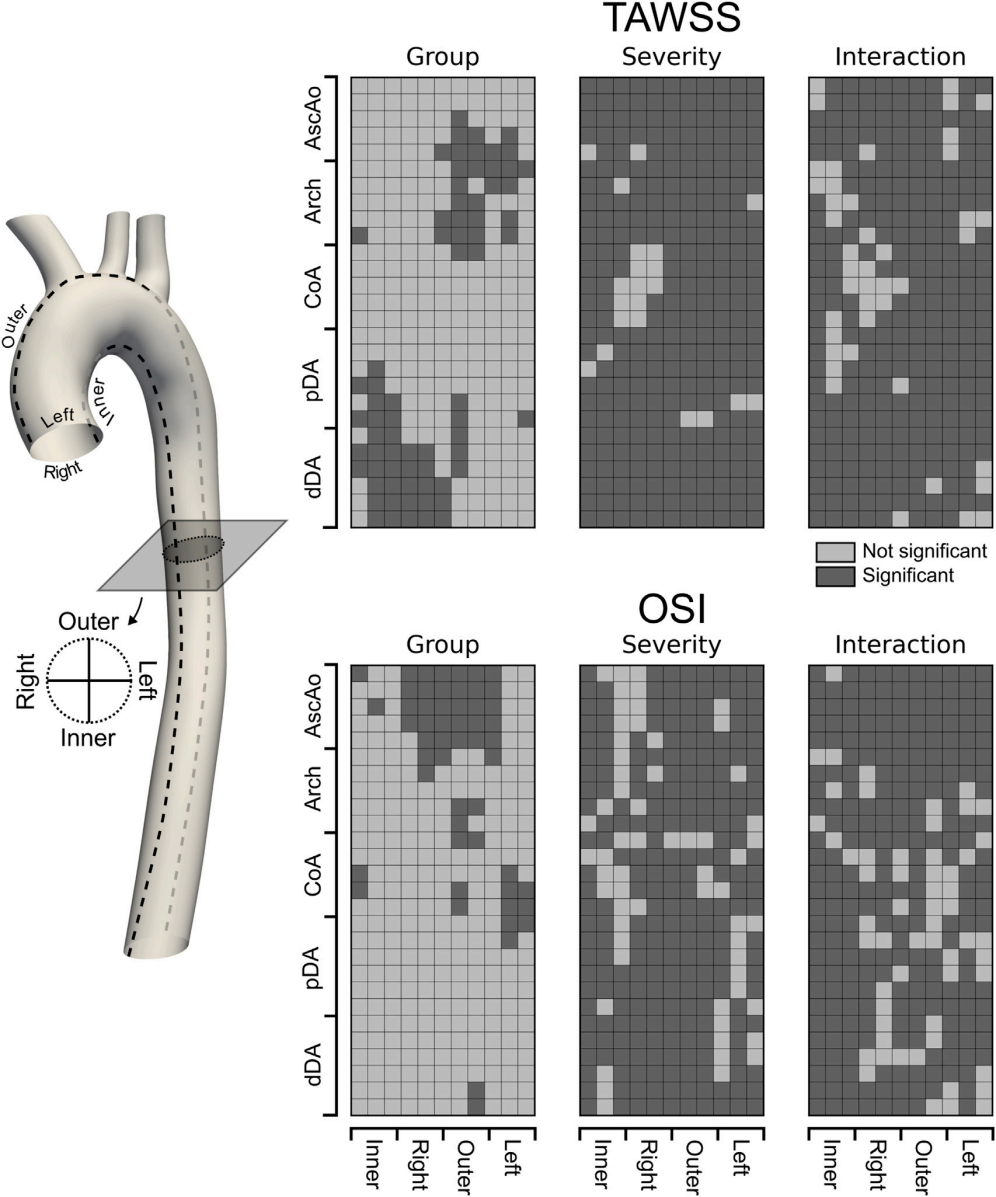
Unwrapped distribution of significant (dark gray) differences in TAWSS and OSI between Repaired and Control groups. A linear mixed effect approach was used to model hemodynamic metrics of interest with designed main effects, i.e., Group, Severity, and the interaction term Group:Severity as fixed effects whereas other patient-specific predictors not included in the current study were lumped into a random effect with one categorical outcome per case. This way, the patient-specific baseline variability in TAWSS and OSI were incorporated into the random effect and accounted for in the statistical analysis. Statistical inferences are made based on a 0.05 level of significance.

The LME model revealed that the group effect (repaired versus control) remained significant in specific regions (18%–24% of the luminal surface of the aortic, regardless of the severity of the re-coarctation. This consistent pattern further highlights that repaired patients consistently experience abnormal hemodynamics. Furthermore, the interaction between group and severity was also significant in most regions, suggesting that the degree of re-coarctation exacerbates these differences but does not alter the fundamental disparity between the groups. Specifically, TAWSS was significantly affected in the AscAo and arch, particularly towards the outer and left regions (Figure 10). Additionally, significant TAWSS patterns were observed on the inner side of the pDA, extending to the inner and right side of the dDA. These findings indicate regions where repaired CoA patients are likely to experience increased shear stress, which can contribute to adverse remodeling and complications. In contrast, OSI exhibited a different pattern of significance. It was primarily affected in the AscAo, particularly on the right outer side. Sparse significant patterns of OSI were also observed in the arch and CoA region, mainly towards the outer and left side of the aorta (Figure 10). These results suggest that the oscillatory nature of shear stress in these regions may play a role in pathological remodeling processes distinct from those driven by TAWSS. In summary, our findings indicate that repaired CoA patients continue to experience adverse hemodynamics compared to healthy controls. The patterns of TAWSS and OSI distribution are distinct between the two groups and are significantly influenced by both the presence of re-coarctation and its severity. These results underscore the importance of considering both the spatial distribution of hemodynamic metrics and the impact of residual or recurrent stenosis when assessing long-term outcomes in CoA patients. The differences in TAWSS and OSI patterns across the aorta may provide insights into the mechanisms driving pathological remodeling and restenosis, especially if correlated with surveillance imaging findings and long-term clinical outcomes. This highlights the potential for improving patient outcomes by integrating CFD simulations and metrics into the routine clinical management of complex, chronic cardiovascular disease such as CoA.

## 4 Discussion

The ideal goal of every CoA repair operation is the restoration of normal aortic anatomy and diameter, with no residual pressure gradient or hemodynamic abnormalities (Stephens et al., 2024). However, patients have anywhere from a 10%–50% incidence of late complications such as restenosis after CoA repair. While population-level risk factors for restenosis have been identified, clinicians cannot yet accurately predict an individual patient’s risk of restenosis (Dias et al., 2020; Stout et al., 2018). As a result, lifetime surveillance for restenosis is mandatory for all patients following CoA repair (Stout et al., 2018; Isselbacher et al., 2022; Baumgartner et al., 2020).

This study was motivated by hypothesis that CFD simulations can likely detect residual hemodynamic abnormalities following CoA repair, especially if care is taken to select clinically-comparable patient cohorts. We found that CoA repair with REEA leaves patients with significantly higher TAWSS in the aortic arch, CoA repair site, and descending aorta. As all simulations were performed under identical starting and boundary conditions, we attribute this finding to the presence of a small but hemodynamically significant residual coarctation following most repair cases.

Our work builds directly upon prior investigations using patients from the same VMR database, which investigated the distributions of TAWSS and OSI in many of the same patient models as in the current study and found that repaired CoA patients may not have normal aortic hemodynamics (LaDisa et al., 2011a; LaDisa et al., 2011b). These prior studies simulated resting and exercise blood flow states in five patients, of whom three were included in the current study (C1, R1, and R5, Figure 1) and, using patient-specific inlet and outlet boundary conditions, showed agreement with clinically measured blood pressure values. For instance, published findings reported TAWSS at the CoA site in C1 was less than R1, but higher than in R5 (2.9, 5.0, and 1.1 Pa-s, respectively), which was consistent with our findings (1.78, 4.2, and 1.5 Pa-s, respectively). Their vessel heatmaps show elevated TAWSS in the CoA region in R1 and low TAWSS in R5, which is also consistent with the findings of the current study. Additionally, they show TAWSS reaching a peak magnitude of 10 Pa-s around the aortic arch and coarctation, as well as distally on the pDA, where the jet impinges on the aortic wall. The consistency of their findings with ours alongside prior validation studies (Gounley et al., 2016b) supports the hypothesis of the current study that 0-pressure outlet simulations provide sufficiently accurate results while adjust the space as per previous lines.

Other indications of hemodynamic abnormalities, such as increased vorticity and power loss at the repair site, have been identified by other investigations (Goodarzi Ardakani et al., 2022). These differences may be due to phenomena such as incomplete resection of the coarcted tissue, or increased turbulence caused by the suture line. Studies of other CoA repair strategies (*i.e.*, stenting) have similarly found evidence of residual stenosis. For example, one investigation of 13 CoA patients who underwent stenting found that the median WSS at the coarctation region dropped from 24.5 to 11.3 Pa following stenting, but remained slightly elevated when compared to an idealized, “virtual” repair that completely restored normal anatomy (median WSS 7.5 Pa) (Goubergrits et al., 2015). This residual difference of roughly 4 Pa is comparable to the difference observed between our control and repaired cohorts.

The relationship between WSS magnitude and cardiovascular pathophysiology is extremely complex and is not yet fully understood (Andersson et al., 2017). Rafieianzab et al. modeled CoA with idealized geometries containing coarctations of 25%, 50%, and 75%, with the additional implementation of deformable walls through a two-way fluid-structure interaction (FSI) model and velocity/pressure boundary conditions at all outlets, but reports surprisingly low TAWSS (5 Pa) with a stenosis severity of 75% (Rafieianzab et al., 2021). Our results showed stenosis severity significantly impacts TAWSS and OSI magnitude, a finding that has not yet been extensively reported on before. The nonlinear relationship between residual coarctation and progressively abnormal shear metrics suggests the possibility of a positive feedback mechanism, whereby trace residual stenoses cause increased wall shear stress, which promotes further stenosis, and so on.

### 4.1 Limitations

The present study should be interpreted in relation to several limitations. This study provides valuable information by using open source patient geometries from the VMR, enhancing reproducibility and accessibility for future research. However, the VMR dataset does not include follow-up imaging or long-term patient outcomes, such as the development of restenosis over time. Future investigations could build on this work by using clinical data sets that include long-term follow-up data and multiple imaging time points, enabling simulations of evolving patient-specific hemodynamics.

Our use of 0-pressure outlet boundary conditions was specifically chosen to isolate the effects of vascular morphology on flow patterns and reduce confounding variables. Although this approach ensures consistency and comparability between patient cohorts, future applications in clinical settings would benefit from tunable boundary conditions to account for downstream vascular adaptation and variability, particularly in cases of severe CoA stenosis, where patient-specific boundary conditions have a more significant effect (Mariotti et al., 2023). For example, incorporating lumped parameter models, such as Windkessel networks, could better capture the dynamics of the vascular response, although these adjustments may introduce additional variability in flow predictions - reported to differ by up to 96% compared to clinical MRI data (Nicole Antonuccio et al., 2021). To balance these considerations, our use of standardized boundary conditions aligns with established practices in computational modeling (LaDisa et al., 2011a), providing a robust foundation for comparative analyses while minimizing potential confounding factors.

Recent comparisons of rigid vs deformable-wall CFD simulations in the ascending thoracic (Calò et al., 2023) and abdominal aorta (Peng et al., 2023) have found that rigid-wall models tend to overestimate aortic TAWSS and underestimate OSI, particularly in the compliant regions of the aorta. However, the site of CoA repair following REEA is known to be significantly less elastic and more rigid than normal aortic tissue (Verhaaren et al., 2001). This suggests that a deformable-wall model would yield even stronger differences, further supporting the robustness of our conclusions. Additionally, deformable-wall models of CoA after EEA also conclude that the presence of residual stenosis is the most significant factor in causing residual hemodynamic abnormalities (Taelman et al., 2016).

Finally, the pronounced variation in TAWSS and OSI patterns observed among repaired patients, compared to controls, appears to stem not only from residual anomalies at the repair site but also from anatomical variability. Factors such as arch angulation and other morphological anomalies, which are absent in the control cohort, significantly influence postoperative shear stress outcomes (Oliver et al., 2009; Goubergrits et al., 2015).

## 5 Conclusion

In this study, we studied aortic hemodynamics in patients following CoA repair and compared them with healthy controls matched in age and sex. Our findings demonstrate significant differences in the magnitude and spatial distribution of TAWSS and OSI between repaired patients and controls, with repaired patients exhibiting hemodynamic alterations related to residual stenosis. These results suggest a potential feedback mechanism in which elevated shear stress may contribute to further stenosis, highlighting a long-term risk for this patient population.

Through systematic variation of the severity of the stenosis, we identified the critical role of geometric factors, particularly the degree and location of the narrowing, in shaping the distribution of shear stress and influencing regions susceptible to pathologic remodeling. These insights emphasize the need to account for anatomic variability in postoperative care, as abnormalities in shear stress driven by anatomy could serve as predictive markers of adverse outcomes.

Overall, this study underscores the potential of CFD simulations as a tool for personalized treatment planning, offering a pathway to optimize patient outcomes and mitigate long-term risks after CoA repair.

## Data Availability

The raw data supporting the conclusions of this article will be made available by the authors, without undue reservation.
